# Long-Term Cost-Effectiveness of Fractional Flow Reserve–Based Percutaneous Coronary Intervention in Stable and Unstable Angina

**DOI:** 10.1016/j.jacadv.2022.100145

**Published:** 2022-12-30

**Authors:** David Hong, Hyunsoo Kim, Hankil Lee, Jin Lee, Juhee Cho, Doosup Shin, Seung Hun Lee, Hyun Kuk Kim, Ki Hong Choi, Taek Kyu Park, Jeong Hoon Yang, Young Bin Song, Joo-Yong Hahn, Seung-Hyuk Choi, Hyeon-Cheol Gwon, Danbee Kang, Joo Myung Lee

**Affiliations:** a Division of Cardiology, Department of Internal Medicine, Heart Vascular Stroke Institute, Samsung Medical Center, Sungkyunkwan University School of Medicine, Seoul, South Korea; b Department of Digital Health, SAIHST, Sungkyunkwan University, Seoul, South Korea; c Center for Clinical Epidemiology, Samsung Medical Center, Sungkyunkwan University, Seoul, South Korea; d College of Pharmacy, Ajou University, Suwon, South Korea; e Department of Clinical Research Design and Evaluation, SAIHST, Sungkyunkwan University, Seoul, South Korea; f Division of Cardiovascular Medicine, Department of Internal Medicine, University of Iowa Carver College of Medicine, Iowa City, Iowa, USA; g Division of Cardiology, Department of Internal Medicine, Chonnam National University Hospital, Gwangju, South Korea; h Department of Internal Medicine and Cardiovascular Center, Chosun University Hospital, University of Chosun College of Medicine, Gwangju, South Korea

**Keywords:** angina pectoris, cost-effectiveness, fractional flow reserve, percutaneous coronary intervention

## Abstract

**Background:**

There are limited studies on the cost-effectiveness of fractional flow reserve (FFR)-based percutaneous coronary intervention (PCI) over angiography-based PCI.

**Objectives:**

The current study sought to evaluate long-term cost-effectiveness of FFR-based PCI compared to angiography-based PCI.

**Methods:**

A cost-effectiveness analysis was conducted using a nationwide cohort that consisted of patients with stable or unstable angina from the National Health Insurance Service (NHIS) and Health Insurance Review and Assessment (HIRA) database in Korea. The cost-effectiveness analysis was also performed by using a decision and Markov model with key values from the United States and the United Kingdom health care systems. Incremental cost-effectiveness ratio (ICER), an indicator of incremental cost on additional quality-adjusted life-years gained by FFR-based PCI, was evaluated.

**Results:**

In the NHIS-HIRA data, FFR-based PCI was used during the index PCI in 5,116 patients (3.8%) among 134,613 eligible patients. FFR-based PCI showed significantly lower risk of all-cause death (5.8% vs 7.7%, *P* = 0.001) and spontaneous myocardial infarction (1.6% vs 2.2%, *P* = 0.022) than the angiography-based PCI at 4 years. In the NHIS-HIRA data, FFR-based PCI gained 0.039 quality-adjusted life-years at a lower cost ($303) than angiography-based PCI, yielding an ICER of −$7,748 during the 4-year follow-up. FFR-based PCI was dominant in the health care system of Korea (ICER = −$7,309), United States (ICER = −$31,267), and United Kingdom (ICER = −$1,341) during a 10-year time horizon. These results were consistently shown in probabilistic sensitivity analyses.

**Conclusions:**

In the current cohort, FFR-based PCI was associated with higher quality of life at a lower cost than angiography-based PCI. FFR-based PCI was cost-effective in patients with stable or unstable angina undergoing PCI.

Measuring the fractional flow reserve (FFR) during an invasive coronary angiography identifies a stenosis that causes myocardial ischemia and enables better selection of patients likely to benefit from a percutaneous coronary intervention (PCI). Previous studies confirmed that FFR was superior in guiding PCI in patients with stable ischemic heart disease than conventional invasive coronary angiography alone.^1^ Even though FFR is a class 1A recommendation in current guidelines for the assessment of intermediate stenosis in stable ischemic heart disease,^2^ the penetration rate of FFR in contemporary practice varies across different health care systems and also remains limited due to multifactorial reasons such as physicians' attitude, knowledge barrier, and environmental barrier (reimbursement, procedural time, or medical cost).^3^ Despite the compelling evidence of a significant reduction in clinical events with FFR-based PCI,^1^^,^^4^ there is limited research on the cost-effectiveness of FFR-based PCI over angiography-based PCI.^5^, ^6^, ^7^, ^8^ Particularly, previous studies have limited generalizability as they were based on either randomized controlled trials (RCTs) with strict inclusion criteria or registry studies that could not thoroughly examine the cost or quality of life. In addition, little is known about whether cost-effectiveness of FFR-based PCI varies across different health care systems.

In this regard, the current study sought to evaluate the long-term cost-effectiveness of FFR-based PCI compared to that of angiography-based PCI using the National Health Insurance Service (NHIS) and Health Insurance Review and Assessment (HIRA) claim database in Korea. In addition, the current study also aimed to evaluate the generalizability of cost-effectiveness of FFR-based PCI across different health care systems using the Markov model.

## Methods

### Study design and data

The current study was performed in 3 phases. Phase 1 was a nationwide cohort study using the NHIS and HIRA nationwide administrative claims database in Korea to generate a comparative prognosis between FFR- and angiography-based PCI.^9^ Phase 2 was an individual patient-level cost-effectiveness analysis of FFR-based PCI over angiography-based PCI using phase 1 data. Phase 3 was the cost-effectiveness analysis of FFR-based PCI over angiography-based PCI based on a decision and Markov model. Key inputs in the model were acquired from phase 1 and 2 data and previous evidence from the nationwide registry as well as RCTs conducted in the United States or the United Kingdom with different health care systems.^10^^,^^11^

The Korean NHIS covers approximately 97% of Koreans, while the 3% of remaining Koreans who cannot afford national insurance are covered by the Medical Aid Program.^12^ Claims submitted for reimbursement to NHIS and Medical Aid Program are reviewed by the HIRA service, a central office in the Korean Ministry of Health. This study was approved by the Institutional Review Board of Samsung Medical Center, and informed consent was waived as deidentified data were used. This study followed the Consolidated Health Economic Evaluation Reporting Standards reporting guideline.^13^

### Study population of phase 1 and 2

In this study, all men and women aged 18 years who underwent PCI for stable or unstable angina at secondary or tertiary hospitals between January 2011 and December 2017 (N = 332,629) were included. The index PCI was defined as the first PCI that was performed during this period. To investigate the underlying comorbidities before the index PCI, patients who had medical records of <1 year prior to the index PCI were excluded from the study (N = 136,522). Patients who had their index PCI for acute myocardial infarction (MI) (N = 61,382), had a history of coronary artery bypass graft surgery (CABG) (N = 6), or were lost to follow-up immediately after the index PCI (N = 106) were also excluded. A total of 134,613 eligible patients were divided into 2 groups according to the use of FFR during the index PCI into FFR-based PCI or angiography-based PCI. Follow-up clinical data were assessed until December 31, 2018.

### Data collection and clinical outcomes

The NHIS-HIRA database contains information about patients' demographics, diagnoses, drug prescriptions, procedures, use of medical devices including pressure wire and type of stents, medical costs, and mortality data. For each patient, the clinical and claims data were longitudinally recorded. As the HIRA routinely audits the claims, such data are considered reliable and used in numerous peer-reviewed publications. Diagnoses were coded using the International Classification of Disease-10th revision. The current study used all-cause death to minimize ascertainment bias in the assessment for the cause of death. Spontaneous MI was defined as receiving PCI or CABG with newly diagnosed MI after the index PCI. Unplanned revascularization was defined as receiving PCI or CABG after 1 month from the index procedure. Details about the codes used to define the study population, past medical histories, medications, procedures, and devices used in PCI, are summarized in Supplemental Table 1.

### Cost-effectiveness analysis using NHIS-HIRA data

In phase 2, we performed individual patient-level cost-effectiveness analyses of FFR-based PCI over angiography-based PCI using phase 1 data in a 4-year time horizon. We calculated the total quality-adjusted life-years (QALYs) in each group by summing the QALYs from the 3 statuses, combining mortality (years of life lost), MI (years lived with MI), and angina (years lived with angina) effects. The utility weight of angina and MI state was obtained from a previous study.^14^ Cumulative medical costs were calculated from the claim records until the occurrence of all-cause death, spontaneous MI, unplanned revascularization, or the end of follow-up period. Costs were limited to medical costs excluding nonmedical and indirect costs.

The cost-effectiveness analysis was represented by the incremental cost-effectiveness ratio (ICER), an indicator of incremental cost on additional QALYs gained by FFR-based PCI. The ICER was obtained from the difference in the cumulative costs of FFR-based PCI and angiography-based PCI divided by the difference in cumulative QALYs between the 2 groups. We applied the willingness-to-pay (WTP) threshold of U.S. $30,000, which was close to the one-time (1x) gross domestic product (GDP) per capita of Korea in 2021, to determine the optimal strategies in the base-case and sensitivity analyses. ICERs lower than the 1x WTP threshold were defined as cost-effective. A strategy that was both less costly and more effective than another was defined as dominant.

We also performed a probabilistic sensitivity analysis (PSA). The incremental cost-effectiveness plane for differences in costs and QALYs and in the ICER was obtained using the bootstrap technique with the percentile method with 25,000 replications. Subgroup analyses were performed by age, congestive heart failure, atrial fibrillation, diabetes mellitus, and clinical diagnosis (stable vs unstable angina).

### Cost-effectiveness analysis using the Markov model

In phase 3, we performed a cost-effectiveness analysis of FFR-based PCI in 3 different health care systems (Korea, U.S., and UK) using a Markov model. We used a 3-state Markov model that simulated angina patients: 1) angina; 2) spontaneous MI; and 3) death. Patients initially allocated to angina could either remain in that same state or transition to spontaneous MI or death in every cycle, according to transition probability (Supplemental Figure 1). Patients who were allocated to spontaneous MI could transit to death over time. Patients who developed unplanned revascularization without MI or death remained in the same state. We simulated subjects with equivalent characteristics as the trial population in phase 1 and modeled the costs and health utilities for 10 years beyond the phase 1 study. A time horizon of 10 years was applied for the model because the starting age for the simulated subjects was 66.8 years, and life expectancy in Korea is around 80 years. Cost and utility data were discounted by an annual rate of 4.5% according to the Korean Guidelines of Methodological Standards for economic evaluation and 3.5% for the cost and utility of U.S. and UK based on previous literature.^13^ We projected the discounted lifetime health care cost by multiplying the number of subjects with the sum of the costs in every health status. QALYs were estimated using the utility values associated with each health status multiplied by the proportion of subjects living in the status. Total QALYs were accumulated from the QALYs values in each cycle. In the U.S. and UK health care systems, the value of $60,000 and $40,000 per QALY was considered a reasonable WTP based on the GDP per capita.

Table 1 summarizes key input parameters including transition probability, utilities, and medical costs used in the cost-effectiveness analysis. Transition probability for the Korean population was acquired from phase 1. Multivariable Cox proportional hazard regression was used to calculate the adjusted HR and 95% CI to compare the risk of clinical events according to the use of FFR during the index PCI. The transition probability for the U.S. and UK populations was obtained from previous evidence of a nationwide registry (Veterans Affairs Clinical Assessment, Reporting, and Tracking Program) and RCT (FAME [Fractional Flow Reserve versus Angiography for Multivessel Evaluation] trial).^10^^,^^11^ The transition probability for death after spontaneous MI was obtained from RCTs (COMPLETE [Complete versus Culprit-Only Revascularization Strategies to Treat Multivessel Disease after Early PCI for STEMI) and FLOWER-MI [Flow Evaluation to Guide Revascularization in Multivessel ST-Elevation Myocardial Infarction] trials).^15^^,^^16^

**Table 1:** Key Inputs in the Model

	Korean Population		U.S. Population		UK Population	
	Value	Source	Value	Source	Value	Source
Transition probabilities						
Spontaneous MI						
Angio-PCI	0.016	HIRA data	0.0079	Parikh et al^10^	0.087	Tonino et al^11^
FFR-PCI	0.013	HIRA data	0.0064	Parikh et al^10^	0.057	Tonino et al^11^
Angio-PCI vs FFR-PCI, HR (95% CI)	0.75 (0.59-0.96)^a^	HIRA data	0.77 (0.47-1.27)	Parikh et al^10^	0.66 (0.42-1.04)	Tonino et al^11^
All-cause death						
Angio-PCI	0.056	HIRA data	0.059	Parikh et al^10^	0.028	Tonino et al^11^
FFR-PCI	0.039	HIRA data	0.028	Parikh et al^10^	0.017	Tonino et al^11^
Angio-PCI vs FFR-PCI, HR (95% CI)	0.80 (0.70-0.91)^a^	HIRA data	0.57 (0.45-0.71)	Parikh et al^10^	0.58 (0.26-1.32)	Tonino et al^11^
Spontaneous MI to death	0.131	HIRA data	0.05	Mehta et al^15^	0.016	Puymirat et al^16^
Unplanned revascularization						
Angio-PCI	0.124	HIRA data	0.034	Parikh et al^10^	0.095	Tonino et al^11^
FFR-PCI	0.128	HIRA data	0.038	Parikh et al^10^	0.065	Tonino et al^11^
Angio-PCI vs FFR-PCI, HR (95% CI)	1.00 (0.92-1.08)^a^	HIRA data	1.04 (0.84-1.28)	Parikh et al^10^	0.68 (0.45-1.05)	Tonino et al^11^
Medical costs, $						
Additional cost of FFR	194	HIRA data	975	Fearon et al^5^^,^^17^	375	Layland et al^18^
Annual cost after angina	1,483	HIRA data	5,225	Bishu et al^19^	406	NHS England
Annual cost after MI	1,524	HIRA data	27,796	Ito et al^20^	1,964	Bravo Vergel et al^21^
Unplanned revascularization without MI	1,473	HIRA data	26,796	Kazi et al^22^	2,652	NHS England^23^
Unplanned revascularization with MI	1,483	HIRA data	36,090	Kazi et al^22^	3,452	NHS England^23^
Death-related cost	6,441	HIRA data	35,818	Kazi et al^22^	1,629	NHS England^23^
Utility						
Established angina	0.79	Kodera et al^14^	0.92	Weintraub et al^24^	0.718	Henriksson et al^25^
Spontaneous MI	0.67	Kodera et al^14^	0.85	Kazi et al, Rinfret et al^22^^,^^26^	0.683	Henriksson et al^25^
Disutility						
Atrial fibrillation with angina	−0.084	Åkerborg et al^27^	-		-	
Atrial fibrillation with MI	−0.145	Åkerborg et al^27^	-		-	
Congestive heart failure	−0.064	Sullivan and Ghushchyan^28^	-		-	
Diabetes with angina	−0.040	Van der Linden et al^29^	-		-	
Diabetes with MI	−0.050	Van der Linden et al^29^	-		-	

CVA = cerebrovascular accident; FFR = fractional flow reserve; HIRA = Health Insurance Review and Assessment; MI = myocardial infarction; PCI = percutaneous coronary intervention.

a Adjusted for age, sex, clinical presentation, hypertension, diabetes mellitus, hyperlipidemia, congestive heart failure, previous CVA, atrial fibrillation, peripheral vascular disease, chronic obstructive pulmonary disease, chronic renal failure, type of stent, number of stents, discharge medications, and medical cost during index admission.

Medical costs for the Korean population were estimated from individual patient-level claim data. Regarding death-related costs, we estimated the medical costs within 6 months before death. To obtain the annual medical cost, cumulative medical costs were divided by the observation period. Medical costs for the U.S. and UK populations were obtained from previous studies.^5^^,^^17^, ^18^, ^19^, ^20^, ^21^, ^22^^,^^23^ The utility weight of angina and MI states in Korea was obtained from the same literature used in phase 2.^14^ For the U.S. and UK populations, the utility weight of angina and MI was obtained from previous studies, which evaluated cost-effectiveness in their health care systems.^22^^,^^24^, ^25^, ^26^

To further assess the intraindividual and parameter uncertainties, PSA by the Monte Carlo Simulation was performed wherein subjects were randomly sampled and simulations repeated 25,000 times to obtain the outcomes. As for the input variable ranges in the simulation, lognormal distribution was used for transitional probabilities, beta distribution for utilities (utility value ranged between 0 and 1), and gamma distribution for costs (costs could not be <0). As a sensitivity analysis, modeling with the different costs and utilities from the different health care systems (U.S. and UK) was performed using input parameters extracted from the previous evidence (Table 1). To identify the impact of changing the key model inputs such as discount rates (0%, 3.5%, and 4.5%) and time horizon (5 and 15 years), a univariate sensitivity analysis was performed. All the modeling processes were performed using R version 4.1.0 (R Foundation for Statistical Computing, Vienna, Austria).

## Results

### Patients and procedural characteristics

Among 134,613 eligible patients who underwent PCI for stable or unstable angina, FFR-based PCI group was used during index PCI in 5,116 patients (3.8%) (Central Illustration upper left panel). The mean age and proportion of female patients were 66.8 ± 10.3 years and 34.1%, respectively. Among the total population, 51.6% and 48.4% of patients presented with stable and unstable angina, respectively, and PCI was performed based on FFR in 4.7% (3,229/69,424) and 2.9% (1,887/65,189) of patients with stable and unstable angina, respectively. Patient and procedural characteristics are presented in Table 2.

**Central Illustration:**
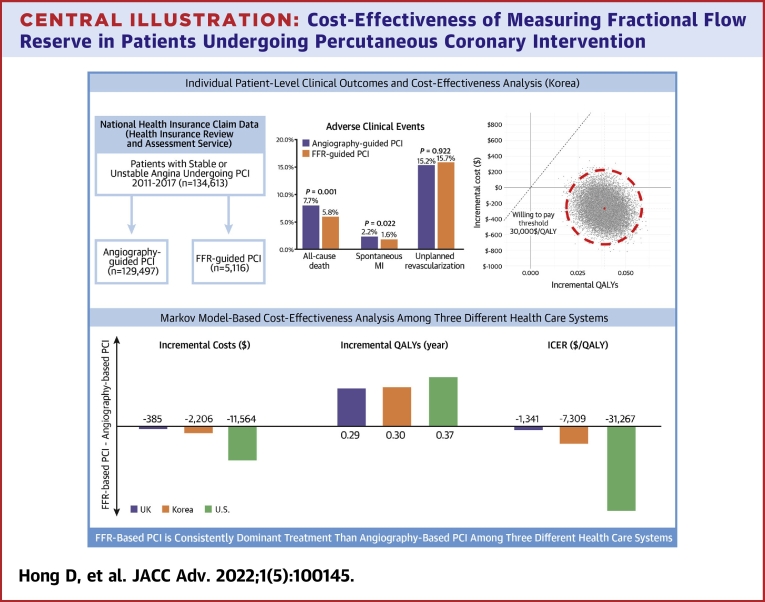
**Cost-Effectiveness of Measuring Fractional Flow Reserve in Patients Undergoing Percutaneous Coronary Intervention** The current study investigated the long-term cost-effectiveness of FFR-based PCI compared to angiography-based PCI in stable or unstable angina patients who underwent PCI using the NHIS-HIRA Claim Database. In this large-scale nationwide data set, FFR-based PCI was shown to achieve better quality of life at a lower cost than angiography-based PCI, which was mainly driven by the lower risk of all-cause death and spontaneous MI. In addition, in a model-based cost-effectiveness analysis, FFR-based PCI was a consistently dominant treatment than angiography-based PCI among 3 different health care systems: Korea, US, and UK. FFR = fractional flow reserve; ICER = incremental cost-effectiveness ratio; MI = myocardial infarction; NHIS-HIRA = National Health Insurance Service and Health Insurance Review and Assessment; PCI = percutaneous coronary intervention; QALY = quality-adjusted life year.

**Table 2:** Patient and Procedural Characteristics of Study Population

	Total (n = 134,613)	Angiography PCI (n = 129,497)	FFR-PCI (n = 5,116)	*P* Value
Demographics				
Age, y	66.8 ± 10.3	66.9 ± 10.3	65.7 ± 10.0	<0.001
Female	45,912 (34.1)	44,353 (34.2)	1,559 (30.5)	<0.001
Clinical diagnosis				<0.001
Stable ischemic heart disease	69,424 (51.6)	66,195 (51.1)	3,229 (63.1)	
Unstable angina	65,189 (48.4)	63,302 (48.9)	1,887 (36.9)	
Cardiovascular risk factors				
Hypertension	96,480 (71.7)	92,735 (71.6)	3,745 (73.2)	0.014
Diabetes mellitus	66,309 (49.3)	63,666 (49.2)	2,643 (51.7)	<0.001
Hyperlipidemia	93,707 (69.6)	89,863 (69.4)	3,844 (75.1)	<0.001
Atrial fibrillation	9,290 (6.9)	8,975 (6.9)	315 (6.2)	0.035
Congestive heart failure	29,697 (22.1)	28,548 (22.0)	1,149 (22.5)	0.495
Chronic renal failure	12,228 (9.1)	11,812 (9.1)	416 (8.1)	0.017
Chronic obstructive pulmonary disease	21,945 (16.3)	21,122 (16.3)	823 (16.1)	0.685
Previous CVA	37,509 (27.9)	36,115 (27.9)	1,394 (27.2)	0.324
Peripheral vascular disease	26,652 (19.8)	25,635 (19.8)	1,017 (19.9)	0.898
Baseline medications				
Aspirin	75,910 (56.4)	72,749 (56.2)	3,161 (61.8)	<0.001
P2Y_12_ inhibitor	55,980 (41.6)	53,799 (41.5)	2,181 (42.6)	0.126
Anticoagulant (warfarin or NOAC)	4,178 (3.1)	4,039 (3.1)	139 (2.7)	0.113
ACEI or ARBs	54,797 (40.7)	52,743 (40.7)	2,054 (40.1)	0.415
Beta-blocker	47,845 (35.5)	45,860 (35.4)	1,985 (38.8)	<0.001
Calcium-channel blocker	52,252 (38.8)	50,153 (38.7)	2,099 (41.0)	0.001
Nitrate	34,368 (25.5)	32,976 (25.5)	1,392 (27.2)	0.005
Statin	80,800 (52.6)	67,758 (52.3)	3,042 (59.5)	<0.001
Ezetimibe	9,088 (6.8)	8,566 (6.6)	522 (10.2)	<0.001
Fenofibrate	5,257 (3.9)	5,029 (3.9)	228 (4.5)	0.041
Medications after index procedure				
Aspirin	123,092 (91.4)	118,286 (91.3)	4,806 (93.9)	<0.001
P2Y_12_ inhibitor	124,521 (92.5)	119,650 (92.4)	4,871 (95.2)	<0.001
Clopidogrel	120,104 (89.2)	115,402 (89.1)	4,702 (91.9)	<0.001
Ticagrelor or prasugrel	15,079 (11.2)	14,480 (11.2)	599 (11.7)	0.251
Anticoagulant (warfarin or NOAC)	8,364 (6.2)	8,079 (6.2)	285 (5.6)	0.056
ACEI or ARBs	87,221 (64.8)	84,094 (64.9)	3,127 (61.1)	<0.001
Beta-blocker	81,809 (60.8)	78,816 (60.9)	2,993 (58.5)	0.001
Calcium-channel blocker	69,738 (51.8)	67,001 (51.7)	2,737 (53.5)	0.014
Nitrate	61,116 (45.4)	59,029 (45.6)	2,087 (40.8)	<0.001
Statin	122,032 (90.7)	117,238 (90.5)	4,794 (93.7)	<0.001
Ezetimibe	26,856 (20.0)	25,527 (19.7)	1,329 (26.0)	<0.001
Fenofibrate	5,325 (4.0)	5,156 (4.0)	169 (3.3)	0.016
Procedure characteristics				
Number of stents used	1.46 ± 0.76	1.46 ± 0.76	1.50 ± 0.79	0.001
Type of device used				<0.001
Drug-eluting stent	122,152 (90.7)	117,424 (90.7)	4,728 (92.4)	
Drug-coated balloon angioplasty	3,344 (2.5)	3,240 (2.5)	104 (2.0)	
Plain old balloon angioplasty	7,250 (5.4)	7,085 (5.5)	165 (3.2)	
Bare metal stent	874 (0.6)	852 (0.7)	22 (0.4)	
Bioresorbable vascular scaffold	993 (0.7)	896 (0.7)	97 (1.9)	

Values are mean ± SD or n (%).

ACEI = angiotensin-converting enzyme inhibitor; ARB = angiotensin receptor blocker; CVA = cerebrovascular accident; FFR = fractional flow reserve; NOAC = non-vitamin K antagonist oral anticoagulant; PCI = percutaneous coronary intervention.

### Cost-effectiveness analysis using NHIS-HIRA data

The median follow-up duration was 3.0 years (IQR: 1.8-4.6 years). At 4 years of follow-up, a total of 7,737 deaths and 2,179 spontaneous MI occurred. The FFR-based PCI group showed significantly lower risk of all-cause death (5.8% vs 7.7%; adjusted HR: 0.798; 95% CI: 0.698-0.913; *P* = 0.001) and spontaneous MI (1.6% vs 2.2%; adjusted HR: 0.751; 95% CI: 0.587-0.959; *P* = 0.022) than the angiography-based PCI group (Central Illustration upper middle panel). There was no significant difference in the risk of unplanned revascularization between the 2 groups (15.7% vs 15.2%; adjusted HR: 0.996; 95% CI: 0.918-1.080; *P* = 0.922) (Table 1, Supplemental Table 2).

**Figure 1:**
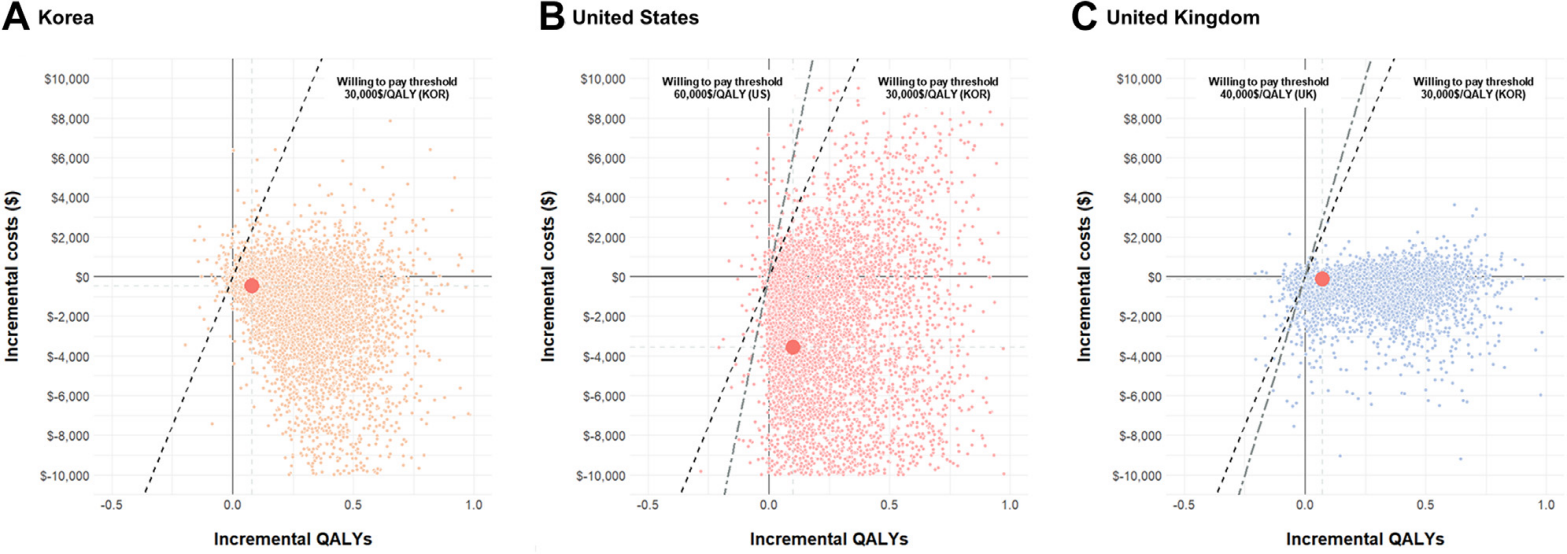
**Incremental Cost-Effectiveness Planes for FFR-Based PCI Compared With Angiography-Based PCI Until 10 Years After PCI Among Different Health Care Systems** This incremental cost-effectiveness planes for **(A)** Korea, **(B)** the United States, and **(C)** the United Kingdom show the impact of FFR-based PCI over angiography-based PCI on the difference in QALYs and accompanying health care–related costs within 10 years after PCI. The result is based on a probability sensitivity analysis (25,000 sample). The average incremental cost-effectiveness ratio (ICER) was presented as a **red dot**. Willingness-to-pay thresholds of $30,000/QALY **(dashed line)** are shown. FFR = fractional flow reserve; PCI = percutaneous coronary intervention; QALYs = quality-adjusted life years.

Regarding the effectiveness, there was a gain of 0.039 QALYs in the FFR-based PCI group compared with the angiography-based PCI group during 4 years of follow-up. The total cumulative cost per patient in the FFR-based PCI group was estimated to be $10,503, about $303 less than that of the angiography-based PCI. Consequently, the ICER was calculated at −$7,748; therefore, FFR-based PCI was a cost-effective alternative during 4 years of follow-up in the NHIS-HIRA database (Table 3). In PSA, FFR-based PCI showed dominant cost-effectiveness than angiography-based PCI (Central Illustration upper right panel, Supplemental Figure 2).

**Table 3:** ICER With FFR-Based PCI Relative to Angiography-Based PCI

Base-Case Analysis	Cost, $		QALYs		Cost-Effectiveness ICER (U.S. $/QALY)
	Total	Incremental	Total	Incremental	
Angiography PCI	10,503	*Reference*	3.037	*Reference*	
FFR-PCI	10,200	−303	3.076	0.039	−7,748

FFR = fractional flow reserve; ICER = Incremental Cost-effectiveness ratio; PCI = percutaneous coronary intervention; QALYs = quality-adjusted life years.

The dominant cost-effectiveness of FFR-based PCI was consistently observed in all subgroups analyzed, particularly FFR-based PCI showed much lower cost and higher QALYs than angiography-based PCI group in patients with congestive heart failure (medical cost −$969 and QALYs 0.07) and diabetes mellitus (medical cost −$858 and QALYs 0.45). In addition, regardless of whether patients presented with stable (ICER −$7,308) or unstable (ICER −$8,805) angina, FFR-based PCI had dominant cost-effectiveness than angiography-based PCI (Table 4).

**Table 4:** Subgroup Analysis of ICER With FFR-Based PCI Relative to Angiography-Based PCI

	Cost, $			QALYs			Cost-Effectiveness ICER (U.S. $/QALY)
	Angio-PCI	FFR-PCI	Incremental	Angio-PCI	FFR-PCI	Incremental	
Age							
<70 y	10,540	9,979	−261	3.090	3.104	0.014	−18,621
≥70 y	10,852	10,551	−301	2.966	3.031	0.065	−4,632
Atrial fibrillation	12,210	11,453	−757	2.584	2.673	0.089	−8,496
Congestive heart failure	12,250	11,281	−969	2.713	2.783	0.07	−13,853
Diabetes mellitus	11,965	11,107	−858	2.808	2.853	0.045	−19,058
Clinical presentation							
Unstable angina	10,419	10,234	−185	3.053	3.074	0.021	−8,805
Stable angina	10,583	10,181	−402	3.022	3.077	0.055	−7,308

FFR = fractional flow reserve; ICER = incremental cost-effectiveness ratio; PCI = percutaneous coronary intervention; QALYs = quality-adjusted life years.

### Cost-effectiveness analysis using a decision and Markov model

After the end of the 10-year simulation, the cumulative mortality and incident MI rates were 46.4% and 11.7% in the angiography-based PCI group and 38.5% and 9.4% in the FFR-based PCI group, respectively. In base-case analysis, FFR-based PCI in Korea showed better quality of life than angiography-based PCI (4.90 vs 4.60 QALYs) in the model. Simultaneously, FFR-based PCIs resulted in lower medical costs ($21,744 vs $23,951) than angiography-based PCIs (Table 5).

**Table 5:** 10-Cycle Simulation Results of ICER With FFR-Based PCI Relative to Angiography-Based PCI

	Cost, $			QALYs			Cost-Effectiveness ICER (U.S. $/QALY)
	Angio-PCI	FFR-PCI	Incremental	Angio-PCI	FFR-PCI	Incremental	
Using transition probability from HIRA data							
Korean population	23,951	21,744	−2,206	4.60	4.90	0.30	−7,309
U.S. population	139,409	127,844	−11,564	5.63	6.00	0.37	−31,267
UK population	8,694	8,308	−385	4.40	4.69	0.29	−1,341
Using transition probability from publications							
U.S. population	120,833	96,011	−24,821	5.78	6.58	0.80	−31,039
UK population	10,755	8.648	−2,106	5.14	5.45	0.31	−6,892

FFR = fractional flow reserve; ICER = incremental cost-effectiveness ratio; PCI = percutaneous coronary intervention; QALYs = quality-adjusted life years.

Although cost-effectiveness of the FFR-based PCI was consistently observed across the 3 different health care systems, the U.S. setting resulted in the highest cost reduction for FFR-based PCIs ($127,844 vs $139,409 in FFR-based PCI and angiography-based PCI groups, respectively), while the UK setting showed the least cost reduction ($8,308 vs $8,694) (Table 5). The QALYs gained from FFR-based PCIs over angiography-based PCIs were also the highest in the U.S. population (0.37) and the lowest in the UK population (0.29). The ICER value in the U.S. setting was the highest at −$31,267, followed by Korea (−$7,309) and UK (−$1,341). Similar results were shown when using transition probability from the previous evidence, and the ICER in FFR-based PCI compared with angiography-based PCI was estimated to be −$31,039 and −$6,892 for U.S. and UK, respectively (Table 5). In the PSA analysis, given the GDP per capita in each country, the likelihood iterations of cost-effectiveness for FFR-based PCIs were 93.5%, 92.3%, and 90.8% for Korea, U.S., and UK, respectively (Figure 1).

The results of the sensitivity analyses by applying 0%, 3.5%, and 4.5% for the discount rate showed ICERs of −$6,825/QALY, −$6,869/QALY, and −$7,309/QALY in Korea; −$30,295/QALY, −$31,267/QALY, and −$31,278/QALY in the U.S.; −$1,340/QALY, −$1,341/QALY, and −$1,341/QALY in the UK, respectively, from the base-case analysis (Supplemental Table 3). In the scenario of change in the time horizon from 5 to 15 years, the ICER decreased from −$7,701/QALY to −$7,296/QALY in Korea, from −$31,912/QALY to −$30,437/QALY in the U.S., and from −$1,359/QALY to −$1,276/QALY in the UK, respectively (Supplemental Table 3).

## Discussion

The current study investigated the long-term cost-effectiveness of FFR-based PCI compared to angiography-based PCI in stable or unstable angina patients who underwent a PCI using the NHIS-HIRA claim database. In this large-scale nationwide data set, FFR-based PCI was shown to achieve better quality of life at lower cost than angiography-based PCI. In addition, in the model-based cost-effectiveness analysis, FFR-based PCI was consistently more cost-effective than angiography-based PCI in 3 different health care systems: Korea, U.S., and UK. Various sensitivity analyses including PSA showed consistent results supporting the cost-effectiveness of FFR-based PCI over angiography-based PCI (Central Illustration).

### Gap of evidence and limited adoption rates of FFR-based PCI

In contemporary practice, the presence of inducible myocardial ischemia is the prerequisite for PCI, and current guidelines support the use of invasive physiologic indexes such as FFR or nonhyperemic pressure ratios for the decision of revascularization in stable ischemic heart disease as a Class Ia recommendation.^2^ These strong recommendations are based on multiple RCTs and large-scaled registries, which consistently demonstrated significantly better clinical outcomes following FFR-based PCIs over angiography-based PCIs.^4^ Due to the limited diagnostic yield of noninvasive functional tests in distinguishing myocardial ischemia originating from epicardial coronary stenosis, invasive physiologic interrogations possess clinical relevance in patients with chronic coronary syndrome. Two recent large observational studies from nationwide cohorts further supported the survival benefit of FFR-based PCI over angiography-based PCI.^10^^,^^30^ In the same line, the current nationwide study also identified significantly lower risk of all-cause mortality and spontaneous MI following FFR-based PCIs in the exclusively revascularized patients for stable or unstable angina. Nevertheless, the global adoption rate of the invasive physiologic assessment with FFR is about <6%.^3^ Similar to that of many other countries worldwide, the adoption rate of FFR in Korea was 3.8% in the current study. Although previous surveys revealed that there are heterogenous reasons for the limited adoption rates of FFR, physicians' attitude, knowledge barrier, and environmental barrier (reimbursement, procedural time, or medical cost) might be some of the most important reasons.^3^ Specifically, studies clarifying the cost-effectiveness of measuring the FFR have been limited.^5^, ^6^, ^7^ In the absence of evidence for the cost-effectiveness of FFR, the additional medical cost due to measuring FFR could act as a hurdle for the use of FFR in decision-making during PCI.

### Cost-effectiveness of FFR-based PCI over angiography-based PCI

To date, the available evidence for the cost-effectiveness analysis in the use of FFR is from RCTs and observational registries.^5^, ^6^, ^7^, ^8^ In the FAME trial, FFR-based PCIs in patients with a multivessel disease provided higher QALYs and lower medical cost than angiography-based PCIs.^5^ In the FAME 2 trial, PCIs rather than medical treatment in patients with FFR ≤0.80 led to improved clinical outcomes as well as quality of life without an increase in cumulative medical costs ($16,737 ± $13,108 vs $16,792 ± $10,139, *P* = 0.94) at 3 years.^6^ Although the PCI group had higher initial medical costs, the medical treatment group had higher follow-up costs which was driven by the higher rate of urgent revascularization. As a result, the ICER for PCI compared with medical treatment was $1,600. The cost-effectiveness analysis from the CVIT-DEFER registry which enrolled 3,228 patients from multiple centers in Japan also presented that FFR guidance resulted in a reduction of total expected medical cost by 137,015 Japan ¥ (yen) per patient (approximately $1,205 per patient).^7^ However, the analysis of the CVIT-DEFER registry focused only on the impact of FFR on medical costs through initial treatment decision without considering the long-term clinical outcomes and medical costs following the initial treatment decision. In addition, the cost-effectiveness analyses from the FAME trials were based on selected patients with strict inclusion criteria, which limited generalizability of the results.

The current study evaluated all-comers who received a PCI from the nationwide cohort which covers the entire Korean population and showed the impact of FFR on long-term cost-effectiveness. Furthermore, as NHIS is the sole insurer in Korea, evaluation of NHIS claim data enabled the capture of all actual health care resource utilization during the patient’s entire life without missing any data. In phase 2 analysis, FFR-based PCIs provided the ICER of −$7,748, supporting that the FFR-based PCI is a dominant treatment that is cost-effective compared to angiography-based PCIs. In phase 3 analysis, the cost-effectiveness analysis using the Markov model also confirmed the long-term cost-effectiveness of FFR-based PCIs across Korean, U.S., and UK health care systems.

### Clinical implications of FFR from economic perspectives

The treatment decision based on FFR has 2 clinical implications. First, FFR provides information to avoid unnecessary PCIs. Second, FFR allows optimal selection of the lesion requiring revascularization. Because the current study exclusively evaluated patients who were treated by PCI, the significantly lower risk of spontaneous MI and all-cause death in the FFR-based PCI group was mainly driven by the second clinical role of FFR. In addition, as patients who were deferred PCI based on insignificant FFR was not included in the current study, FFR-based PCIs had higher cost at index admission due to the cost of pressure wire and hyperemic agents. Nevertheless, the additional cost of measuring FFR during the index procedure was offset, and the total cumulative medical costs were lower in the FFR group based on the significantly lower risk of spontaneous MI and all-cause death during 4 years of follow-up. The significantly lower risk of hard clinical outcomes raised QALYs in FFR-based PCIs, consequently, FFR-based PCI was a dominant treatment strategy over angiography-based PCI.

In the subgroup analyses, FFR was more cost-effective in the high-risk patients for ischemic heart disease, such as diabetes mellitus, or in patients who share common risk factors with ischemic heart disease, such as atrial fibrillation or heart failure. These findings suggest that the benefit of appropriate lesion selection through FFR at the index procedure would be greater for high-risk patients than for low-risk patients. Furthermore, among 3 different health care systems, cost-effectiveness of FFR-based PCI was more remarkable in the U.S. health care system, which has higher medical cost than Korean or UK system. This difference in cost-effectiveness was mainly attributable to relatively lower medical costs incurred when adverse clinical events were treated in Korea or UK, whose public health care system regulates medical costs. These results emphasize the greater potential benefit of FFR in patients with multiple comorbidities and in health care systems with higher medical costs.

### Study Limitations

Some limitations should be acknowledged. First, since the cost-effectiveness analysis was based on a retrospective cohort, susceptibilities related to the study design, such as measured or unmeasured confounding factors, were also inherent. Second, the results of the current study were strongly dependent on key input values such as transition probability, medical costs, and utilities, which were obtained through limited research. However, both NHIS-HIRA data-based and the model-based analyses showed consistent results, and multiple sensitivity analyses also support the cost-effectiveness of FFR-based PCI over angiography-based PCI. Third, the current study assumed common key input values for all individuals, irrespective of their own baseline characteristics, such as age and sex. Fourth, the current results could not be applied to health-care systems other than Korea, U.S., or UK. Fifth, the current data could not represent the use of nonhyperemic pressure ratios. Finally, and importantly, this study is only able to show that FFR-PCI is cost-effective in patients selected for this strategy. We were unable to study differences in angiographic or procedural characteristics between groups. It is not possible to determine if FFR-PCI should be applied more broadly. Patients in the FFR-PCI group may have had different coronary characteristics, such as severe stenosis, for which FFR would be unnecessary.

## Conclusions

Patients treated with FFR-based PCI achieved a higher quality of life at a lower cost than those treated with angiography-based PCI. FFR-based PCI was cost-effective in patients with stable or unstable angina undergoing PCI. Current results support the contemporary guidelines that, in patients selected for FFR-guided PCI, there are better clinical outcomes and the approach is cost-effective.

PERSPECTIVES **COMPETENCY IN MEDICAL KNOWLEDGE:** Measuring FFR during invasive coronary angiography identifies a stenosis that causes myocardial ischemia and enables better selection of patients likely to benefit from PCI.

**COMPETENCY IN PATIENT CARE:** Previous studies confirmed that FFR was superior in guiding PCI in patients with a stable ischemic heart disease than conventional invasive coronary angiography alone. Despite compelling evidence for a significant reduction in clinical events with FFR-based PCIs, there is limited research on the cost-effectiveness of FFR-based PCIs over angiography-based PCIs.

**TRANSLATIONAL OUTLOOK:** In this large-scale nationwide data set, FFR-based PCI was shown to achieve better quality of life at lower cost than angiography-based PCI. In a model-based cost-effectiveness analysis, FFR-based PCI was consistently more cost-effective than angiography-based PCI in 3 different health care systems: Korea, U.S., and UK. Current results support the contemporary guidelines that, in patients selected for an FFR-guided PCI, there are better clinical outcomes and the approach is cost-effective.

## Funding support and author disclosures

Dr Joo Myung Lee received an institutional research grant from Abbott Vascular, Boston Scientific, Philips Volcano, Terumo Corporation, Zoll Medical, and Donga-ST. All other authors have reported that they have no relationships relevant to the contents of this article to disclose.
