# Supplementary material for: Long-Term Cost-Effectiveness of Fractional Flow Reserve–Based Percutaneous Coronary Intervention in Stable and Unstable Angina
Source: JACC Adv. 2022 Dec 30;1(5):100145. doi: 10.1016/j.jacadv.2022.100145 (PMC11198057; doi:10.1016/j.jacadv.2022.100145)
Supplement: Supplemental Tables 1-3, Figures 1 and 2 [file mmc1.docx]

**Supplemental Table 1. Codes Used to Define Study Population, Past Medical Histories, Medications, Procedures, and Devices Used in PCI**

| Diagnosis | ICD-10 Codes |
| --- | --- |
| Ischemic heart disease | I20, I21, I25.6 |
| Myocardial infarction | I21 |
| Angina |  |
| Stable ischemic heart disease | Codes excluding I20.0 among I20, I25.6 |
| Unstable angina | I20.0 |
| Hypertension | I10-13, I15 |
| Diabetes mellitus | E10-14 |
| Hyperlipidemia | E78 |
| Atrial fibrillation | I48 |
| Congestive heart failure | I11.0, I13.0, I13.2, I25.5, I42.0, I42.5-9, I43, I50, I97.1 |
| Chronic renal failure | I12.0, I13.1, N03.2-7, N05.2-7, N18-19, N25.0, Z49, Z94.0, Z99.2 |
| Chronic obstructive pulmonary disease | J43-46 |
| Previous CVA | G45-46, H34.0, I60-69 |
| Peripheral vascular disease | I70-71, I73.1, I73.8-9, I77.1, I79.0, I79.2, K55.1, K55.8-9, Z95.8-9 |
| **Medications** | **ATC Codes** |
| Antiplatelet |  |
| Aspirin | A01DA05, B01AC06, B01AC30, B01AC56, C07FX02-04, C08CA01, C10BX01-02, C10BX04-06, C10BX08, C10BX12, M01BA03, M03BA53, N02AJ02, N02AJ07, N02AJ18, N02BA01, N02BA51, N02BA71, N02BE51, R05X, R06AA57 |
| Clopidogrel | B01AC04, B01AC30, C08CA01 |
| Ticagrelor | B01AC24 |
| Prasugrel | B01AC22 |
| Anticoagulant (Warfarin or NOAC) | B01AA03, B01AE07, B01AF01-03 |
| ACEI or ARBs | C09AA01-16, C09BA01-09, C09BA12-13, C09BA15, C09BB02-07, C09BB10, C09BB12, C09BX01-05, C09CA01-10, C09DA01-10, C09DB01, C09DB02, C09DB04-09, C09DX01-07, C10BX04, C10BX06-07, C10BX10-18 |
| Beta blocker | C07AA01-03, C07AA05-07, C07AA12, C07AA14-17, C07AA19, C07AA23, C07AA27, C07AB01-14, C07AG01-02, C07BA02, C07BA05-07, C07BA12, C07BA68, C07BB02-04, C07BB06-07, C07BB12, C07BB52, C07BG01, C07CA02, C07CA03, C07CA17, C07CA23, C07CB03, C07CB02-03, C07CB53, C07CG01, C07DA06, C07DB01, C07FB02-03, C07FB07, C07FB12-13, C07FX01-06 |
| Calcium channel blocker | C07FB02, C07FB03, C07FB07, C07FB12, C07FB13, C08CA01-16, C08CA51, C08CA55, C08CX01, C08DA01, C08DA02, C08DA51, C08DB01, C08EA01-02, C08EX01-02, C08GA01-02, C09BB02-07, C09BB10, C09BB12, C09DB01-09, C09DX03, C10BX03 |
| Nitrate | C01DA08, C01DA14, C01DA58, C01DX12, C01DX16, C05AE02 |
| Statin | A10BH51-52, C10AA01-08, C10BA01-09, C10BX01-18 |
| Ezetimibe | C10BA02, C10BA05, C10BA06, C10AX09, C10BA10 |
| Fenofibrate | C10AB05, C10AB11, C10BA03, C10BA04, C10BA09 |
| **Procedures and Devices** | **Korea HIRA EDI Codes** |
| Percutaneous coronary intervention | M6551-6654, M6561-6567, M6571, M6572, O1876-1877 |
| Coronary artery bypass graft surgery | O1640-1642, O1647-1649, OA640-642, OA647-649 |
| Pressure wire | J6081 |
| Type of devices |  |
| Drug-eluting stent | J5083, J8083 |
| Drug-coated balloon angioplasty | J4080, J8080 |
| Plain old balloon angioplasty | M6551-6552, O1871-1872 |
| Bare metal stent | J5231, J5603, J8231 |
| Bioresorbable vascular scaffold | J5084 |

Abbreviations: ACEI, angiotensin converting enzyme inhibitor; ARB, angiotensin receptor blocker; ATC, anatomical therapeutic chemical; EDI, electronic data interchange; CVA; cerebrovascular accident; HIRA, Health Insurance Review and Assessment service; ICD-10, International Classification of Diseases-10^th^ revision; NOAC, non-vitamin K antagonist oral anticoagulant.

**Supplemental Table 2. Clinical Outcomes Between Angiography-based and FFR-based PCI**

| **Clinical Events** | **Total**  **(N=134,613)** | **Angio-based PCI**  **(N=129,497)** | **FFR-based PCI**  **(N=5,116)** | **HR, 95% CI, and P value** | |
| --- | --- | --- | --- | --- | --- |
|  |  |  |  | **Unadjusted** | **Multivariable**^*^ |
| **All-Cause death** | 7,737 (7.6%) | 7,532 (7.7%) | 205 (5.8%) | 0.724 (0.633-0.828), P<0.001 | 0.798 (0.698-0.913), P=0.001 |
| **Spontaneous MI** | 2,179 (2.2%) | 2,115 (2.2%) | 64 (1.6%) | 0.778 (0.609-0.993), P=0.044 | 0.751 (0.587-0.959), P=0.022 |
| **Unplanned revascularization** | 15,733 (15.2%) | 15,147 (15.2%) | 586 (15.7%) | 1.013 (0.934-1.098), P=0.752 | 0.996 (0.918-1.080), P=0.922 |

Abbreviations: CI, confidence interval; FFR, fractional flow reserve; MI, myocardial infarction, HR, hazard ratio; PCI, percutaneous coronary intervention.

**Supplemental Table 3. Univariable Sensitivity Analysis Following Variation of Key Model Inputs**

|  | **Cost, $** | | | **QALYs** | | | **Cost-effectiveness**  **ICER (US $/QALY)** |
| --- | --- | --- | --- | --- | --- | --- | --- |
|  | **Angio-PCI** | **FFR-PCI** | **Incremental** | **Angio-PCI** | **FFR-PCI** | **Incremental** |  |
| **Discount rates** |  |  |  |  |  |  |  |
| **0%** |  |  |  |  |  |  |  |
| Korean population | 29,420 | 26,668 | -2,752 | 5.68 | 6.09 | 0.40 | -6,825 |
| US population | 297,618 | 271,278 | -26,340 | 8.65 | 9.52 | 0.87 | -30,295 |
| UK population | 10,592 | 10,108 | -483 | 5.20 | 5.56 | 0.36 | -1,340 |
| **3.5%** |  |  |  |  |  |  |  |
| Korean population | 23,951 | 21,744 | -2,206 | 4.81 | 5.13 | 0.32 | -6,869 |
| US population | 139,409 | 127,844 | -11,564 | 5.63 | 6.00 | 0.37 | -31,267 |
| UK population | 8,694 | 8,308 | -385 | 4.40 | 4.69 | 0.29 | -1,341 |
| **4.5%** |  |  |  |  |  |  |  |
| Korean population | 23,951 | 21,744 | -2,206 | 4.60 | 4.90 | 0.30 | -7,309 |
| US population | 131,789 | 120,919 | -10,870 | 5.38 | 5.73 | 0.35 | -31,278 |
| UK population | 8,241 | 7,879 | -362 | 4.21 | 4.48 | 0.27 | -1,341 |
| **Time horizon** |  |  |  |  |  |  |  |
| **5 years** |  |  |  |  |  |  |  |
| Korean population | 10,736 | 9,898 | -838 | 2.91 | 3.02 | 0.11 | -7,701 |
| US population | 61,688 | 57,577 | -4,111 | 3.49 | 3.62 | 0.13 | -31,912 |
| UK population | 4,217 | 4,081 | -135 | 2.73 | 2.83 | 0.10 | -1,359 |
| **15 years** |  |  |  |  |  |  |  |
| Korean population | 37,768 | 34,132 | -3,635 | 5,57 | 6.07 | 0.50 | -7,296 |
| US population | 219,991 | 200,855 | -19,135 | 6.92 | 7.55 | 0.63 | -30,437 |
| UK population | 12,956 | 12,331 | -624 | 5.41 | 5.90 | 0.49 | -1,276 |

Abbreviations: FFR, fractional flow reserve; ICER, Incremental Cost-effectiveness ratio; PCI, percutaneous coronary intervention; QALYs, quality-adjusted life years; UK, United Kingdom; US, United States.

To identify the impact of changing key model inputs such as discount rates, time horizon, utilities of asthma exacerbation, mortality rate due to hospitalization, or costs, a univariate sensitivity analysis was performed with plausible extremes based on available evidence.

**Supplemental Figure 1. Markov Model**

**
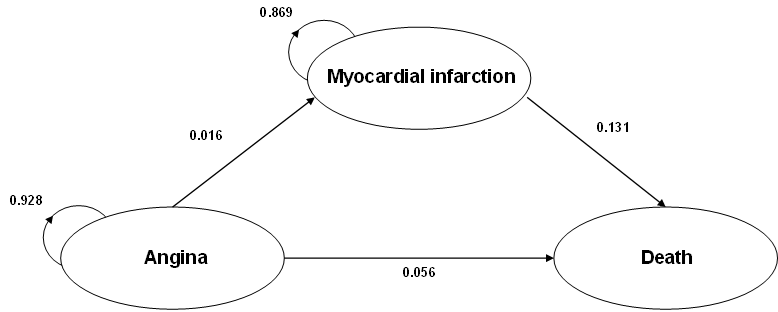
**

Patients transition from 3 different states represented as arrows based on transition probability: (1) angina; (2) spontaneous myocardial infarction; (3) death.

**Supplemental Figure 2. Incremental cost-effectiveness plane for FFR-based PCI compared with angiography-based PCI until 4 years after PCI.**

**
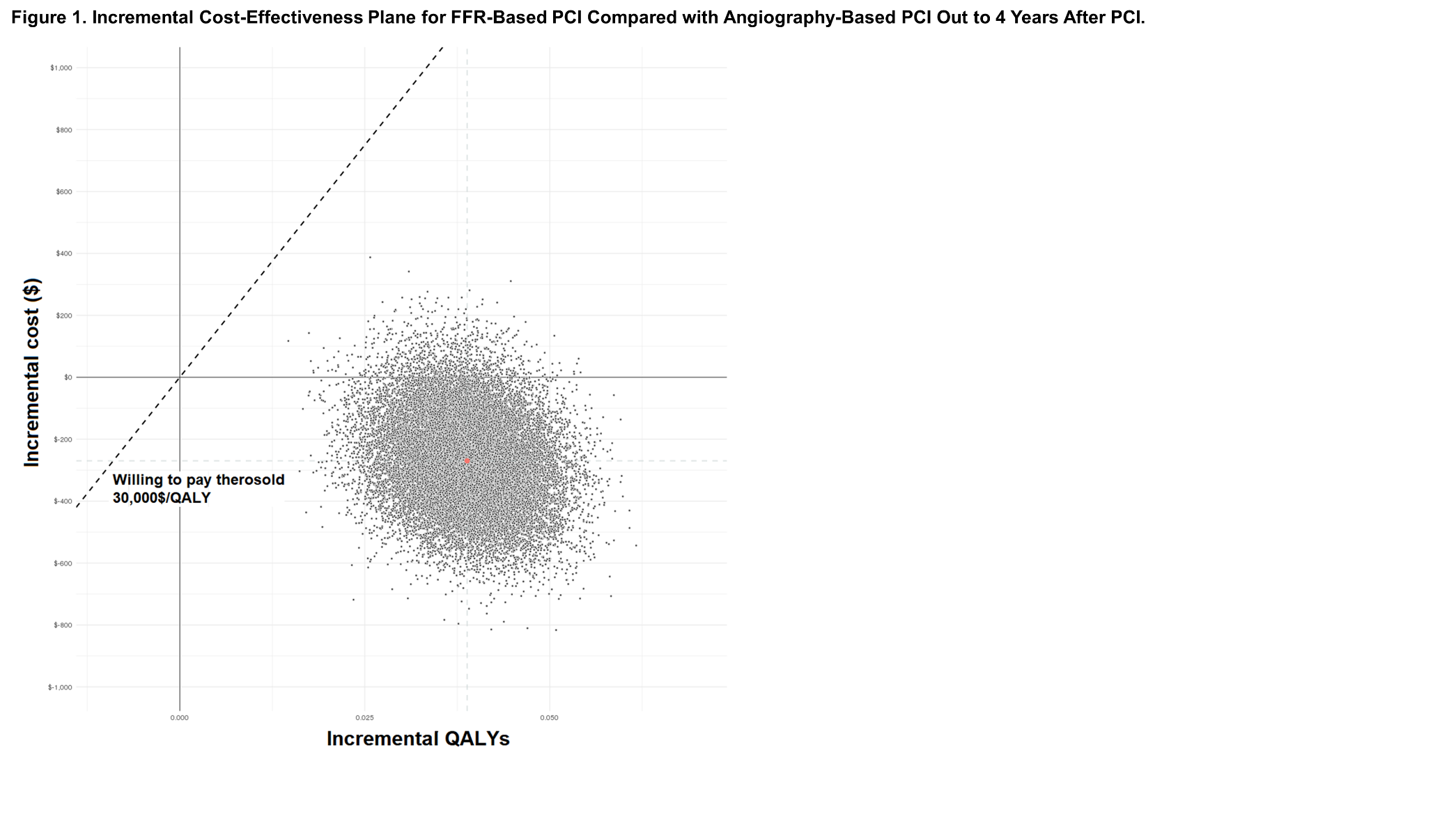
**

Replications of the incremental cost-effectiveness of FFR-based PCI compared with angiography-based PCI are shown. Incremental cost-effectiveness plane presented the impact of FFR use during PCI, as compared to angiography-based PCI, on the difference in QALYs and accompanying healthcare-related costs during 4 years of follow-up. Each of the 25,000 points represents the result of 1 bootstrap replication. The difference in cumulative costs is displayed in the vertical axis, and the difference in QALYs is displayed on the horizontal axis. The average incremental cost-effectiveness ratio (ICER) was presented as a red dot. Willingness-to-pay thresholds of $30,000 per QALY added (dashed line) are indicated in the plane.

Abbreviations: FFR, fractional flow reserve; PCI, percutaneous coronary intervention; QALYs, quality-adjusted life years.
